# Three-dimensional genome architecture and emerging technologies: looping in disease

**DOI:** 10.1186/s13073-017-0477-2

**Published:** 2017-09-30

**Authors:** Arpit Mishra, R. David Hawkins

**Affiliations:** Division of Medical Genetics, Department of Medicine, Department of Genome Sciences, Institute for Stem Cell and Regenerative Medicine, University of Washington School of Medicine, Seattle, WA 98195-5065 USA

## Abstract

Genome compaction is a universal feature of cells and has emerged as a global regulator of gene expression. Compaction is maintained by a multitude of architectural proteins, long non-coding RNAs (lncRNAs), and regulatory DNA. Each component comprises interlinked regulatory circuits that organize the genome in three-dimensional (3D) space to manage gene expression. In this review, we update the current state of 3D genome catalogues and focus on how recent technological advances in 3D genomics are leading to an enhanced understanding of disease mechanisms. We highlight the use of genome-wide chromatin conformation capture (Hi-C) coupled with oligonucleotide capture technology (capture Hi-C) to map interactions between gene promoters and distal regulatory elements such as enhancers that are enriched for disease variants from genome-wide association studies (GWASs). We discuss how aberrations in architectural units are associated with various pathological outcomes, and explore how recent advances in genome and epigenome editing show great promise for a systematic understanding of complex genetic disorders. Our growing understanding of 3D genome architecture—coupled with the ability to engineer changes in it—may create novel therapeutic opportunities.

## Background

**Fig. 1:**
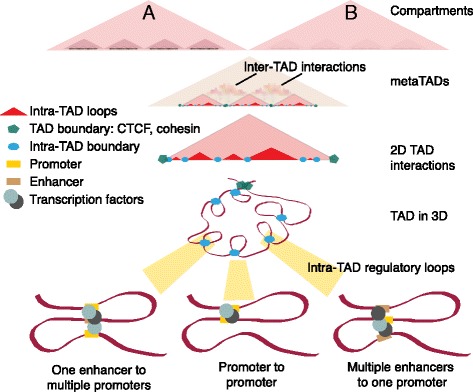
Hierarchical chromatin organization. *Top tier*: higher-order compartments A and B, where A is an active compartment and B is an inactive or densely packed compartment (*beige-colored top-most triangles*). Moving downward, topologically associated domains (*TADs*) are organized into increasingly higher-resolution structures. *Second tier*: representative metaTAD structure (*gray-colored triangle*), where many TADs together form one metaTAD. Inter-TAD interactions, while more sparse, can be detected. *Third tier*: TADs (*light pink triangle*) consist of numerous intra-TAD regulatory loops (*small red triangles* in TADs). These regulatory loops are major governing factors for differential transcriptional output. In tiers 1–3, *triangles* represent higher-frequency contacts of the three-dimensional (*3D*) genome shown in two dimensions (*2D*). *Tier four* illustrates how a TAD may look in 3D, comprising intra-TAD regulatory loops. Representative examples of regulatory loops are also shown: one enhancer to multiple promoter interactions, promoter–promoter interactions, and multiple enhancers to one promoter interactions. TAD boundaries are marked by the CTCF–cohesin complex (*green pentagon*). Intra-TAD elements likely consist of different transcription factors (*light green circles*) and long non-coding RNA (*dark gray circles*)

Chromosomal organization and compaction is an evolutionarily conserved feature. Large genomes need to be condensed into the minute 3D space of the nucleus in a systematic manner in order to retain functional capacity to interact with the gene regulatory machinery. Such a robust yet dynamic looping architecture facilitates fine-tuning of gene expression by mediating the contacts between distantly located *cis*-regulatory elements. Hence, spatial DNA organization performs a secondary role as a global regulator of gene expression. The 3D architecture of DNA is hierarchical in nature (Fig. 1). The fundamental architectural units develop from interactions of DNA and histone octamers in the form of nucleosomes, which leads to the formation of chromatin fibers. Chromatin fibers are further looped and facilitate regulatory interactions by forming insulated neighborhoods of regulatory loops, where multiple regulatory loops assemble to form chromosomal domains or topologically associated domains (TADs), on the scale of 500 kilobases (kb) to 1 megabase (Mb) [1–3]. The currently favored model suggests that TADs are formed by looping long stretches of DNA via anchor proteins such as the CCCTC-binding factor (CTCF)–cohesin complex [4–6]. Intra-TAD looping, including regulatory loops, primarily facilitates dynamic gene expression, while a minor fraction of gene regulatory looping also crosses TAD boundaries, known as inter-TAD regulatory loops (Fig. 1) [7, 8]. TAD sizes are organized for enhancer-to-gene target functionality, and physical insulation of interactions within TADs indicates that regulatory functionality is further optimized at the sub-TAD level [9].

Associations of similar TADs form the next level of organization, known as chromosomal compartments. Referred to as A/B compartments, A is associated with the open euchromatin of transcriptionally active states and is found internally in the nucleus, while B is associated with closed chromatin that lacks significant histone modification enrichment and transcriptionally silent states, found at the nuclear periphery [6]. TAD organization also follows a hierarchical tree-like structure [9, 10]. TADs interact to form metaTADs; however, the interacting TADs are not always nearest neighbors, which suggests that hierarchical complexity rather than linear distance governs chromosomal organization [10]. At the highest order of organization each chromosome occupies a distinct chromosomal territory [11–13].

To sustain dynamic genome architectural changes cells deploy multiple tools. Major architectural proteins include CTCF, cohesin, lamins, the Mediator complex, and transcription factors (TFs). CTCF, an 11-zinc-finger-domain DNA-binding protein, is one of the most studied architectural proteins [14]. Approximately 15% of CTCF-binding sites are found at TAD boundaries, and most other binding sites are involved in intra-TAD regulatory loop interactions [15]. CTCF binds at CpG-containing motifs, and DNA methylation at these sites can abrogate CTCF binding [16, 17]. Cohesin, which was first shown to facilitate sister chromatid cohesion [18, 19], aids looping through interactions with other architectural proteins and protein complexes such as CTCF and Mediators. In the absence of cohesin, TADs remain intact, but their overall packing is affected, resulting in increased inter-TAD interactions and reduced intra-TAD interactions [13, 20]. The Mediator complex helps form the pre-initiation complex at active genes through its interactions with transcriptional machinery bound to *cis*-regulatory elements both proximal and distal to genes, such as promoters and enhancer elements, respectively [19]. This form of regulatory looping brings together enhancers and target promoters, which can be kilobases to megabases apart in the linear DNA sequence (Fig. 1).

In addition to TADs often being found internally in nuclear compartments, there is another component to chromosomal architecture that is near the nuclear periphery or nuclear lamina. These chromosomal architectural units are known as lamina-associated domains (LADs). LADs mainly consist of gene-depleted regions and are part of B compartments [1, 6, 10]. They are mainly associated with lamin B, lamin A, and its alternative spliced product lamin C [21]. Finally, lncRNAs serve key roles in mediating chromosomal architecture (for review see [22]), as illustrated by one of the best characterized lncRNAs, *XIST*, which regulates the compaction of the inactive X chromosome by creating one compact mega domain and preventing TAD formation. The interplay of these factors gives dynamicity to the genome and influences the position of the mutational landscape.

Defining genome architectural mechanisms of diseases will provide novel avenues for disease treatment and management. An advanced understanding of the human genome sequence and GWASs has led to the discovery that the majority of disease-associated mutations or genomic rearrangements lie in gene-desert (non-coding) regions of the genome. Unlike pathogenic mutations in coding regions, the molecular mechanisms of disease for these kinds of genomic aberrations cannot be as easily connected to underlying target genes. A genome architectural context for these variations may provide an understanding of how non-coding mutations influence pathology by altering *cis*-regulatory sequences such as enhancers, silencers, and insulators. These local or global changes in DNA topology may explain molecular mechanisms for many disorders, including cancer and developmental disorders.

The field of 3D genome organization is rapidly progressing and is already revealing the 3D structure to have a role in disease biology [13, 23, 24]. In this review, we provide a brief overview of recent technical advances and a further update on how 3D genomics is impacting our understanding of disease. Techniques such as single-cell Hi-C, capture Hi-C (CHi-C), Hi-C chromatin immunoprecipitation (HiChIP), and proximity ligation-assisted chromatin immunoprecipitation followed by sequencing (PLAC-seq), when combined with GWASs and other omic, microscopy, and CRISPR-based approaches, are helping elucidate the mysteries of chromosomal organization-mediated gene regulation (Table 1). Moreover, we have highlighted the importance of publicly available 3D genome maps for linking regulatory mutations to target genes, and how disease phenotypes mediated by architectural changes can be reconstructed in model systems using genome editing to gauge underlying mechanisms. These novel combinatorial methodologies have already successfully identified pathomechanisms for various diseases.

**Table 1:** Commonly used terminologies

Terminology	Definition
Euchromatin	Chromatin that contains loosely packed nucleosomes. Usually represents transcriptionally active sites in the genome, including regulatory elements
Heterochromatin	Chromatin that is densely packed with nucleosomes. Usually represents transcriptionally silent site in the genome
DNase I hypersensitive sites (DHSs)	Nucleosome-free regions of chromatin that are mostly found at enhancers and promoters. Largely indicative of transcription factor binding
Enhancer elements	Enhancers are sequences of DNA that enhance gene expression by being bound by transcription factors and looping to interact with gene promoters. These elements are located on the same chromosome (*cis*-regulatory) and can be near promoters or megabases away
Super-enhancer	Group of multiple enhancers located within 12 kb of each other, which are bound by an array of transcription factors and marked by acetylation
Temp enhancer	A novel class of *cis*-regulatory elements whose disruption leads to temporary loss of target gene expression, which is eventually regained
Human-gained enhancer	Putative novel enhancer-like elements gained in the human lineage, discovered from brain Hi-C data
Purifying selection	Negative selection in which deleterious alleles are selectively removed through evolution
Gene desert	Large genomic regions that are devoid of genes, but may harbor many disease-causing variants and distal regulatory elements
Promoter interacting regions (PIRs)	PIRs are broadly defined as distal regulatory elements interacting with promoters via looping interactions
Frequently interacting regions (FIREs)	FIREs are regional groups of putative enhancer-like elements that interact with each other and many promoters
Population average ensemble structure	During Hi-C experiments in bulk, cells are present in multiple growth stages; thus, they exhibit multiple 3D architectural landscapes. In bulk Hi-C, different architectural landscapes are captured and this is called population average ensemble structure
Haplotype phasing	Deciphering haplotype block structures for polymorphic sites using genotype data. This is traditionally done computationally to determine if variants are on the same allele. Hi-C provides an experimental means of determining if variants reside on the same allele
Combinatorial indexing	Method that tags DNA within intact nuclei in each cell with successive rounds (combinatorial) of nucleic acid barcodes for adapting to different genomics application such as transcriptomics, Hi-C and chromatin accessibility for single-cell studies, without the need for isolating single cells physically

*3D* three-dimensional, *DHSs* DNase I hypersensitive sites, *HiC* genome-wide chromatin conformation capture, *FIREs* frequently interacting regions, *kb* kilobases, *PIRs* promoter interacting regions, *Temp* temporarily phenotypic

## Approaches to studying disease and 3D genome architecture

Approaches to understanding 3D genome architecture can be divided into two major categories. The first comprises microscopy and fluorescent in situ hybridization (FISH), methods that allow visualization of looping interactions. With the advent of super-resolution and cryo-electron microscopy, resolution limits have improved such that 11-angstrom structures for 30-nanometer fibers can be achieved [25]. The second category covers chromosomal conformation capture (3C)-based approaches, which leverage proximity ligation to “capture” looping interactions [13, 26]). There are now a number of 3C-based methods (for recent reviews see [26–28]; Table 2). The most relevant to this review is Hi-C and its derivatives, which in principle can capture all interactions genome-wide, connect *cis-*regulatory elements harboring disease variants with their target genes, and provide insight on large structural rearrangements in the genome.

**Table 2:** List of genome architectural methods

Technique	Most applicable scenario and/or advantages	Limitations	Relevant example(s)	Suitable computational pipeline(s)
DNA-centric view of genome architectural methods				
Chromosome conformation capture (3C) [92]	Interrogating looping interactions between single gene locus to single regulatory locus (one locus to one locus)	Not suitable for high-throughput identification of novel looping interaction	Association of causal variants from GWASs in 16p13 to *DEXI* gene in type 1 diabetes and multiple sclerosis [93]	Not required
Circular chromosome conformation capture on chip (4C) [94]	Exploring all possible interactions with a single clinically relevant locus (one locus to all loci)	Limited throughput	Association of regulatory SNP with target genes [95]	FourCseq [96]
Circular chromosome conformation capture combined with sequencing (4C-seq) [32]	Exploring all possible interactions with a single clinically relevant locus (one locus to all loci)	GC content or length of interacting fragment may introduce PCR bias	Chromosomal rearrangement detection [97]	FourCseq [96]
Chromosome conformation capture carbon copy (5C) [98]	Studying interactions between many chromosomal loci with many interacting regions across the genome (many loci to all loci)	Complicated primer/probe design can introduce amplification bias. Occasionally misses weak long-range contacts	Determined interaction profiles at pilot promoter regions in ENCODE project [2]. X-chromosome 5C study provided first evidence of topologically associated domains (TADs) [99]	HiFive [100]
Genome-wide chromatin conformation capture (Hi-C) [11] or its variant (in situ Hi-C) [6]	Circumstances where extensive chromatin reorganization occurs (i.e., stem cell differentiation), in which it is important to understand interactions between all parts of the genome (all loci to all loci). The most extensively used genome architectural method	Insensitive method for probing local intra-TAD interactions (<40 kb) unless performed at very high resolution	Genome-wide TAD distributions [1, 6]. Three-dimensional (3D) architectural changes during mitosis [101]. Determination of chromosomal translocations [102]	Methods are primarily divided into: (1) quality control and mapping; (2) domain calling; (3) visualization; and (4) 3D modeling. These methods have been extensively reviewed earlier [46, 47]
Tethered conformation capture (TCC) [103]	Proximity ligation step performed on solid substrate, thus reducing random intermolecular ligation (all loci to all loci)	Although more specific, proximity ligation occurs outside the cell, and thus some native cell context may be lost. Biotinylation step may require optimization	Originally applied to B-cell line, but intended for direct clinical/diagnostic applications	Accompanied by a novel method for TCC data analysis [103]
Genome-wide chromatin conformation capture with DNase I digestion (DNase Hi-C) or targeted DNase Hi-C [36]	Improves on Hi-C restriction-enzyme-mediated resolution limits, and thus is most suitable for higher-resolution architectural studies (all loci to all loci)	DNase I treatment may digest bait-targeted region; thus, tiling probes designed across the region are needed for targeted DNase Hi-C	Targeted DNase Hi-C was used to investigate chromatin architecture at many lncRNA loci	Analysis pipelines are similar to Hi-C methods
Genome architectural mapping (GAM) [66]	First ligation-free method for investigating *cis*-interactions in an unbiased manner. Moreover, it can capture three-way interactions more effectively than Hi-C	Time and specialization required to individually section and dissect out nuclei. Cell asynchrony and heterogeneity affect overall outcome	Uses thin tissue sample slices, which can be applied to frozen clinical tissue samples	GAMTools: specialized automated pipeline [66]
DNA-centric view of genome architectural methods with target enrichment				
Chromosome conformation capture (3C) coupled with oligonucleotide capture technology (capture-C) [34] or next generation (NG-capture-C) [104]	Delineating interaction profiles for many chromosomal loci in a single experiment without introduction of PCR bias and without missing weak long-range interactions (many loci to all loci)	Initial capture-C data suffered from insufficient depth and captured some non-specific interactions. NG-capture-C overcomes these limitations and provides higher sensitivity and resolution	Initially found complex patterns of HIF response by defining chromatin architecture at multiple HIF-bound enhancer and promoter sites [105]. Can be applied to SNP-specific chromatin interaction profile generation	Capture-C analyzer and capture-C oligo design tools available on github [104]
Targeted locus amplification (TLA) [106]	Little requirement of prior sequence knowledge. Most suitable for studying chromosomal rearrangements, single nucleotide variants (SNVs), transgene integration sites, and haplotyping at large genomic intervals. Entire restriction fragments are sequenced, unlike 4C-seq where only ends of fragments are analyzed (many loci to all loci)	Potential for applying to purified genomic DNA or formalin-fixed paraffin embedded material, but current protocol is limited to cells only	Used for haplotyping at *BRCA1* locus. Identified uncommon SNVs and indels. Identification of *ApoE* transgene locus, viral integration sites, and used to study chromosomal rearrangement for *MLL* gene	TLA analysis pipeline details in [107]
Targeted chromatin capture (T2C) [108]	Provides affordable diagnostic tools with restriction enzyme resolution to understand domain and compartments at clinically relevant site. Can be applied to many regions of the genome simultaneously (many loci to all loci)	Output limited to preselected regions. Does not perform well at repeat regions	Was used to validate architecturally well-characterized mouse β-globin and human *H19*/*IGF* loci	No specialized pipeline. Uses mainly well-known tools such as BWA, Samtools, and BEDtools
Hi-C coupled with RNA bait capture probes (CHi-C) [82]	Provides high-resolution *cis-*interactome data at clinically relevant loci such as regulatory elements, single nucleotide polymorphisms (SNPs) from GWASs, TAD boundaries or promoters. Important tool for connecting GWAS outcomes to target genes (many loci to all loci)	Difference in hybridization of RNA probes may introduce enrichment bias. RNA probe location is restricted due to restriction enzyme sites and requires tilling of probes, which increases cost	Identification of three cancer-associated gene deserts in *cis*-interactome [55]. *Cis*-interactome at 14 colorectal-cancer-risk-associated loci [83]. Many other clinically relevant examples discussed in the review	CHiCAGO tools [109]
Promoter capture-Hi-C (p-CHi-C) [7]	Similar to CHi-C, but RNA enrichment baits target all promoters (many loci to all loci)	Similar limitations to CHi-C	A detailed catalogue of 22,000 promoter interactions where autoimmune- and hematological-disorder-related SNPs are significantly enriched [84]	CHiCAGO tools [109]
Promoter-anchored chromatin interaction (HiCap) [35]	Similar approach to CHi-C but uses a 4-bp cutter restriction enzyme for improved resolution (many loci to all loci)	Similar limitations to CHi-C	Promoter-anchored interactions for 15,905 promoters in mouse embryonic stem cells (mESCs)	CHiCAGO tools [109]
DNA-centric view of single-cell genome architectural methods				
Single-cell genome-wide chromatin conformation capture (single-cell Hi-C) [41]	Can delineate cellular heterogeneity at architectural level. Overcomes limitation of population ensemble average structure from bulk Hi-C (all loci to all loci at single-cell level)	Can be technically more challenging than bulk Hi-C. Data from multiple, individual cells are likely needed for a useful interpretation	Single-cell Hi-C has been used to understand architectural heterogeneity for Th1 cells, cell cycle transition and during oocyte to zygotic transition [110, 111]	Single cell Hi-C Pipeline (scell_hicpipe) [41]
Single-cell combinatorial indexing Hi-C (sciHi-C) [42]	Probes cellular heterogeneity by using combinatorial indexing, thus eliminating requirement of single-cell separation using fluorescence-activated cell sorting. Provides rapid scaling for large number of cells. Technically feasible to use for clinically important tissue samples (all loci to all loci at single-cell level)	Comparatively new method; may require optimization compared to bulk Hi-C	sciHi-C data for more than 10,000 single cells was reported. Yet to be explored clinically, but has potential for application to important diseases such as cancer where cellular heterogeneity plays crucial role	Single-cell combinatorial indexing Hi-C pipeline on github [42]
Protein-centric view of genome architectural methods				
Chromatin interaction analysis-end tag sequencing (ChIA-PET) [112]	To understand the protein-specific chromatin interactome. Important in identifying chromatin architectural roles for proteins (many loci to all loci)	Requires known/target protein of interest, similar to chromatin immunoprecipitation followed by sequencing (ChIP-seq). Protein may not bind directly to DNA but bind in complex	Used for studying chromatin architecture mediated by estrogen receptor α binding [112] and CTCF [15]. Applied to diseases such as cancer, can provide an understanding of how changes in the binding of these factors alter 3D genome structure and gene expression	ChIA-PET2 data analysis pipeline [113]
Hi-C chromatin immunoprecipitation (HiChIP) [37]	Protein-centric view of genome architecture similar to ChIA-PET but more sensitive and requires fewer cells (many loci to all loci)	As above	Identified genome-wide cohesin-mediated looping interactions [37]. Can be used to determine disease-altering looping structure for specific architectural proteins	Uses Hi-C Pro for data processing; Fit-Hi-C, Mango, and Juicer for contact interaction calls; and MACS2 for peak calls [114–118]
Proximity ligation assisted chromatin immunoprecipitation (PLAC-seq) [38]	Protein-centric view of genome architecture similar to ChIA-PET, but more sensitive and requires fewer cells (many loci to all loci)	As above	Generated improved maps of promoter–enhancer interactions in mESCs using H3K4me3 mark. Can be used in place of CHi-C methods and does not require probe design/acquisition	PLAC-seq data analysis pipeline [38]

*3C* chromosome conformation capture, *3D* three-dimensional, *4C* circular chromosome conformation capture on chip, *4C-seq* circular chromosome conformation capture combined with sequencing, *5C* chromosome conformation capture carbon copy, *bp* base pairs, *capture-C* chromosome conformation capture coupled with oligonucleotide capture technology, *ChIA-PET* chromatin interaction analysis-end tag sequencing, *CHi-C* Hi-C coupled with RNA bait capture probes, *ChIP-seq* chromatin immunoprecipitation followed by sequencing, *DNase Hi-C* genome-wide chromatin conformation capture with DNase I digestion, *GAM* genome architectural mapping, *GWAS* genome-wide association study, *Hi-C* genome-wide chromatin conformation capture, *HiCap* promoter-anchored chromatin interaction, *HiChIP* Hi-C chromatin immunoprecipitation, *kb* kilobases, *mESC* mouse embryonic stem cell, *NG-capture-C* next-generation capture-C, *p-CHi-C* promoter capture-Hi-C, *PLAC-seq* proximity ligation assisted chromatin immunoprecipitation, *sciHi-C* single-cell combinatorial indexing Hi-C, *SNP* single nucleotide polymorphism, *SNV* single nucleotide variant, *T2C* targeted chromatin capture, *TAD* topologically associated domain, *TCC* tethered conformation capture, *TLA* targeted locus amplification

In Table 2, we briefly summarize the most suitable applications and limitations of genome architectural methods (for a detailed review see [29–31]) and list suitable computational pipelines for analysis of these genome architectural data.

Hi-C-based approaches are at the forefront of guiding our understanding of TAD-level organization and loop formations. HiC-based 3D maps of the genome continue to improve in resolution. High-resolution architectural maps for nine different cell types [6] further reduced the average size of TAD organization to around 185–200 kb—a substantial reduction from early studies [1, 2]. Improving the resolution of 3D maps provides a framework for fine-mapping interactions of novel distal disease variants and their target genes, which could be of therapeutic interest.

### CHi-C and similar directed Hi-C approaches

Deconvolution of the genetic basis of diseases requires high-resolution interaction maps for all genic elements. For now, reliable identification of intra-TAD interactions—such as regulatory loops—from Hi-C data remains a challenge due to the complexity of Hi-C libraries and the substantial cost for the sequence depth required to achieve statistically significant interactions. While targeted locus amplification (TLA) and targeted chromatin capture (T2C) techniques (Table 2) promise to provide *cis*-regulatory information for a limited subset of clinically relevant loci at a substantially reduced cost, CHi-C and subsequent variations for improving throughput were developed to enrich for regions of interest across the genome from complex Hi-C libraries by utilizing specific probes against preselected bait regions in a manner reminiscent of exome capture.

Similar to circular chromosome conformation capture combined with sequencing (4C-seq) before it [32, 33], CHi-C parallelizes the one-to-all approach while reducing the cost of standard Hi-C, and has the potential to map all distal interactions with target regions such as gene promoters. Each capture-based approach also aims to improve upon the resolution of interacting fragments of the genome by applying different DNA cutting enzymes. A similar method, namely chromosome conformation capture coupled with oligonucleotide capture technology (capture-C) [34], wherein genome-wide 3C libraries are fragmented and enriched using oligonucleotide capture technology, was developed to interrogate *cis*-interactions for 450 promoters. The study reported that promoter interaction probability is highest within 600 kb up- or downstream of the transcription start site. Similarly, Hi-C capture uses the 4-bp restriction enzyme MboI for improved resolution, and when applied to generate promoter-anchored interactions for 15,905 promoters revealed 71,984 distal interacting regions in mouse embryonic stem cells (mESCs) [35]. Such techniques may be helpful for validating disease-associated variants that modify promoter interactions in mouse models, or to find putative interactions within human syntenic regions. To further overcome resolution limits posed by the usage and availability of restriction sites across the genome, genome-wide chromatin conformation capture with DNase I digestion (DNase Hi-C) was developed [36]. Coupling DNase Hi-C with capture probes for 998 long intervening noncoding RNA (lincRNA) promoters provided approximately 1-kb resolution of interacting sites in human embryonic stem cells (hESCs) and in the chronic myelogenic leukemia cell line K562. Expansion of this method to all human promoters has the potential to provide the highest-resolution maps to date for interacting distal regulatory elements. This is of great importance when considering disease variants that may localize to distal regulatory elements. Target genes are likely regulated by multiple regulatory elements, and fine-mapping the interacting region of the genome that harbors the regulatory element and disease-associated variant is key to determining the likelihood of causality for the variant through dysregulation of gene expression. Below we highlight several examples of how regulatory variant and target gene interactions are being confirmed.

## Emerging methodologies for genome architecture and disease

Understanding how epigenetic modifications and architectural proteins help define chromatin looping is of immense value for advancing our understanding of genome architecture, and specific techniques have been developed to focus on these associated molecular modifiers. For example, HiChIP provides a protein-centric view of genome architecture by coupling ChIP-seq with Hi-C, and has identified genome-wide cohesin-mediated looping interactions [37]. A similar method, PLAC-seq, targets H3K4me3 histone marks to generate improved maps of promoter–enhancer interactions in mESCs defined by this chromatin modification [38]. Although designed for the same goals as chromatin interaction analysis by paired-end tag sequencing (ChIA-PET), HiChIP and PLAC-seq require less starting material, which improves library complexity and signal-to-noise ratios. These methods also work independently of multiple probes, unlike CHi-C methods, and thus can be less costly, and ideally prevent probe-binding biases. HiChIP and PLAC-seq should provide useful insights for diseases arising from mutations affecting epigenetic modifiers, TFs, TF-binding loci, and architectural proteins. Below we highlight examples focused on architectural proteins, but future applications could include applying HiChIP or PLAC-seq to numerous chromatin modifiers that are the targets of epigenetic therapies [39], as mutations in several of these modifiers likely alter the 3D genome structure in addition to chromatin structure.

Hi-C and CHi-C provide information about population-averaged ensemble structures, as they are performed on millions of cells. (Further pros and cons for capture-based Hi-C methods have been reviewed elsewhere [40].) However, there is a growing appreciation for the heterogeneity found among cells in normal as well as diseased tissues, and that such architectural heterogeneity can be revealed at the single-cell level [41]. Combinatorial indexing of Hi-C has been developed to distinguish single-cell heterogeneity in 3D architecture more effectively. The addition of combinatorial indexing (Table 1) to Hi-C eliminates the need for cell separation and throughput increases exponentially with each round of indexing [42]. This approach can effectively determine chromosomal inversions, deletions, and rearrangements occurring at the single-cell level within a tumor sample, providing insights into intratumoral evolution with the potential to identify therapeutically relevant drivers or other selective mutations within the lesion.

Hi-C catalogues are a valuable resource for understanding disease variants. Integrating 3D genomic data with genetic data and applying polymer modeling approaches with Hi-C catalogues may recapitulate architectural effects of disease variants [8, 43, 44] and can serve as reference 3D genome maps for clinically relevant samples—useful for generating testable hypotheses toward therapeutic opportunities. Extensive datasets, protocols, and software for understanding dynamic 3D genome data can be explored at the 4D Nucleome Portal [45], and a range of computational tools are now available for managing and interrogating chromosomal capture datasets, particularly those generated from Hi-C (Table 2) [46–48].

### Using genome-editing tools to understand genome architecture in health and disease

Modern genome-editing applications such as CRISPR-Cas9 [49] have been employed to study genome architecture and can be broadly classified into three categories: visualization of chromatin dynamics using Cas9 variants; generation of disease models by genome editing; and high-throughput screening for regulatory elements and their effects on chromosomal looping dynamics. Each holds the potential to provide unique insight into disease manifestations.

A limitation to “C”-based techniques is their inability to provide real-time data on chromatin dynamics. Hence, CRISPR-based live cell imaging has been developed to visualize chromatin dynamics by simultaneously tracking multiple genomic loci. CRISPR-based multicolor labeling systems can be used to monitor multiple loci simultaneously. This is achieved through different fluorescently labeled, orthologous catalytically inactive “dead” Cas9 (dCas9) proteins [50], or CRISPRainbow [51], which utilizes engineered single-guide RNA (sgRNAs) such that multiple fluorescent tags can be attached to each guide RNA. Monitoring the localization of these tags can permit tracking of genome-wide topological changes in real time [51] and can be used to validate Hi-C data, epigenetic-related architectural changes, and mutation-associated topology changes. Similarly, CASFISH is a FISH variant based on a HaloTag-fused dCas9 that binds fluorescent ligands. Various fluorescent ligands can, therefore, be targeted to different loci in assorted combinations to monitor looping. Although CASFISH has not been applied to live cell imaging, it is technically feasible [52] as another method to track dynamic looping in real time. CRISPR-enabled visualization of real-time architectural changes at selected loci may further define deleterious effects of chromosomal aberrations on architectural arrangement and could have clinical implications in conditions where deletions, duplication, copy number variations, and inversions are causal, as it will provide a means to dissect dysregulation caused by structural variations, and provide a basis for future diagnostic or prognostic developments. These real-time looping visualization techniques can be introduced into induced pluripotent stem cell disease models to pinpoint temporal or molecular stages in which altered regulatory loops or structural elements begin to affect cell function.

CRISPR-Cas9 methodologies can be used for targeted genome editing to reproduce disease-specific mutations in cell lines or model organisms. This has thus far largely been achieved through deletions of coding regions. Currently, efforts are underway to delete or modify regulatory elements to understand non-coding disease mutations. Recently, Lupiáñez and colleagues showed that topological chromosomal changes resulting in malformation of human limbs can be elegantly recapitulated in the mouse using CRISPR-Cas9 [53]. CRISPR-Cas9 tools have also been employed to confirm that disruption of architectural boundaries in nonmalignant cells leads to activation of proto-oncogenes [54]. Similarly, genome editing was used to understand the effects of CTCF-binding site (CBS) orientation and their looping pattern by inverting the CBS orientation, which led to altered expression of target genes due to change in looping direction [55]. Considering the versatility of CRISPR-Cas9, a multitude of disease models based on genetic and structural variants are sure to follow in both animal and cellular systems.

Because 3D genome architecture and epigenetic changes are intertwined, targeted modification of epigenetic factors could be instrumental to understanding structural changes induced by such events. A large repertoire of dCas9 proteins linked with different epigenetic modifiers broadens the applicability of these enzymes to epigenome editing. Using dCas9 fused with either the TET1 or DNMT3A methylation-modifying enzyme demonstrates the impact of DNA methylation on distal enhancer regulation, CTCF-mediated looping, and influencing changes in overall DNA architecture [56]. Similarly, forms of dCas9 facilitating transcriptional activation have been used to modify epigenomic landscapes, which may in turn change the architectural landscape of extremely long-range promoter–promoter interactions [57–59]. High-throughput epigenome editing techniques have been developed, as well as screening methods for phenotypes resulting from epigenetic changes [60–62]. Fulco and colleagues applied genome-wide, high-throughput CRISPR interference (CRISPRi) screens to explore novel enhancers surrounding the *MYC* and *GATA1* loci, which influence proliferative activity in a leukemia model—demonstrating the utility of this technique to explore *cis-*regulatory influence on disease-relevant phenotypes [63]. Similar epigenomic regulatory element screening can be performed using dCas9-KRAB for repression and dCas9-p300 for activation. For example, a recent study used CRISPR-Cas9-based epigenetic regulatory element screening (CERES) to identify novel regulatory elements of the β-globin and *HER2* loci in human cancer cell lines [64]. More recently, the CRISPRi approach was paired with combinatorial barcoding and single-cell RNA-sequencing (RNA-seq), termed Mosaic-seq, and demonstrated the importance of defining epistatic interactions between enhancer elements to fully understand their effect on target gene expression [65]. Targeting enhancers in these assays assumes an impact on target genes as measured by RNA-seq, which may not be true for all enhancers (i.e., temporarily phenotypic enhancer (Temp) enhancers; see below) [60]. These high-throughput approaches will continue to be developed towards genome-scale interrogation and will further shed light on the capacity in which distal elements drive looping structure. Although CRISPR-Cas9-based genome-editing approaches are promising, they still suffer from off-targeting. To address this, multiple strategies such as ribonucleoprotein (RNP)-based orthologues of Cas9 and modifying sgRNAs are being investigated.

Distal regulatory elements are brought into spatial proximity with their target genes through smaller, likely intra-TAD loops often referred to as regulatory loops. The deletion and repression of distal regulatory elements are expected to influence the architectural landscape. A number of studies discussed below suggest putative complex regulatory three-way interaction—wherein multiple enhancers can regulate a common promoter [43], or multiple promoters converge at a common enhancer [66], or promoter–promoter interactions—wherein enhancer function is attributed to a promoter [67–69]. Such complex regulatory loops are presumably necessary for accurate control of gene expression, and therefore probably differ across cell types or within a disease context, such as overexpression of oncogenes in cancer cells. dCas9 epigenetic modifiers are a valuable novel technology for robust and high-throughput modeling of 3D architectural-based pathologies.

## Genome architecture dysregulation and disease pathogenesis

It is now understood that many disease-associated mutations reside in non-coding regions of the genome; however, primary sequencing has been limited to date for defining precise pathological mechanisms for these non-genic variants. Evidence exists that mutation type and rate are dependent on primary DNA sequence as well as tertiary DNA arrangement (for review, see [70]). It is notable that mutation rates across the genome vary [71], and that chromatin architecture can be highly variable through different developmental stages and between cell types. As a result, the fluctuating mutation rate is strongly related to changes in DNA accessibility [72], and it was recently reported that the mutational contours of cancer are largely determined by the chromatin landscape of the cell type of origin [73]. It is well established that regulatory elements overlap with DNase I hypersensitive sites (DHSs). DHSs are known to be under purifying selection [74]. Interestingly, the mutation rate within DHSs also varies between cell states and types; that is, pluripotent cells and immortalized cells show higher mutation rates in DHSs when compared with the DHSs of differentiated cells [74]. This observed heterogeneous mutational spectrum across cell types aligns well with the cell-type specificity of intra-TAD interactions. Thus, it is feasible that differences in mutation rate may provide a dynamic adaptive mutation range to regulatory elements for fitting in different regulatory circuits. Moreover, mutations are the basis of disease, and this interrelation with chromatin states points toward the importance of 3D genome architecture for a detailed understanding of pathogenesis. For example, phenotypes including limb malformations and proto-oncogene activation have been observed arising from detrimental mutations that disrupt existing TAD boundaries or create spurious new TAD interactions [53, 54] (Table 3).

**Table 3:** Architectural changes and disease

Architectural component	Disease phenotype or mutation effect	Underlying cause or architectural change	References
CTCF	Silencing of tumor suppressor *XAF1*	Hypermethylation of CTCF-binding site near *XAF1* promoter	[119]
CTCF	Illegitimate enhancer access of *PDGFRA* and its overexpression	Hypermethylation of CTCF-binding site due to IDH mutation and disruption of TAD boundary	[120]
CTCF	Human limb malformation	Altered TAD structure surrounding *WNT6/IHH/EPHA4/PAX3* due to deletion, duplication or inversion in CTCF boundary element	[53]
CTCF-cohesin	Activation of proto-oncogenes in T-cell acute lymphoblastic leukemia	Microdeletion of insulated boundary and aberrant access of enhancer to oncogene	[54]
Cohesin loading factor NIPBL in 50% of cases	Cornelia de Lange syndrome	*NIPBL* mutation leads to chromatin decompaction in gene-rich regions. Chromatin architectural dysregulation suspected, but no direct evidence	[19, 121]
MED12	X-linked mental retardation Opitz Kaveggia syndrome	Recurrent mutation R961W in *MED12,* which affects its interaction with ncRNA *a-1* and ncRNA *a-3*, and, therefore, likely disruption of regulatory loops mediated by MED12 and ncRNAs	[122, 123]
Lamin A	Hutchinson–Gilford Progeria syndrome	Point mutation in lamin A, loss of H3K27me3, which in turn leads to global loss of spatial chromatin structure at the nuclear lamina	[124–126]
Long non-coding RNA (lncRNA) *CCAT1-L*	Colorectal cancer	This lncRNA is transcribed from an 8q24 gene desert and interacts with CTCF to form looping structuresat the *MYC* locus, leading to overexpression	[127]
lncRNA *CISR-ACT*	Brachydactyly type E	Translocation-mediated disruption of *cis*-interactions between a lncRNA and the parathyroid hormone-like hormone (*PTHLH*) gene, reducing its expression level	[128]

*lncRNA* long non-coding RNA, *ncRNA* non-coding RNA

Disruption of factors regulating genome architecture can cause deleterious changes in genome topology. For example, deletions, duplications, or changes in the epigenetic landscape that lead to aberrant binding of CTCF or associated architectural proteins and lncRNAs in turn alter TAD structure. The master regulator of DNA architecture, CTCF, has been implicated in a multitude of diseases. Targeted therapies related to these disruptions are still lacking but are of high clinical interest for cases in which hypermethylation in cancer cells disrupts CTCF binding, with available demethylating agents having the potential to restore CTCF binding (see Table 3 for representative examples).

### Generation of 3D genome catalogues and integrative analysis

As the majority of significant non-coding variants from GWASs fall within DNase hypersensitive regions such as enhancers, silencers, or insulators [75, 76], determining how distal, non-coding regulatory variants impact gene expression and in turn have pathological consequences is important. High-resolution interaction maps will prove essential in this effort and have already revealed novel insights into the complexity of disease genetics and *cis-*regulation. Here, we highlight several recent studies.

Recent 3D architectural studies in the brain have emphasized their potential for elucidating complex mechanisms of neuropsychiatric disorders that are not fully understood (for review see [77, 78]). In brain function, long-term potentiation (LTP) and synaptogenesis are very dynamic events that need to be regulated by rapid gene expression changes. Therefore, when the impulse for LTP or synaptogenesis is present, rapid dynamic looping may load transcriptional-machinery-rich enhancers to the promoter for rapid gene regulation. These kinds of neuronal-impulse-induced architectural movements were observed previously for the brain-derived neurotrophic factor (*Bdnf*) locus in mice and satellite DNA loci [79, 80]. Detailed Hi-C maps for cortical and germinal brain regions identified increased promoter–enhancer interactions compared with other tissues [81]. The authors found that novel human-gained enhancers showed significant overlap with lineage-specific lncRNAs and 108 significant schizophrenia-associated variants. This study and others like it have important implications for disorders and diseases outside the nervous system.

The influence of modifications to distal regulatory elements spans tissue types as well as disease types. An extensive study of 21 different cell and tissue types determined thousands of frequently interacting enhancer regions (FIREs) using Hi-C maps [43]. These FIREs are tissue specific in nature and most correspond to active enhancers, as defined by chromatin state. Among all the FIREs detected, 354 are classified as super-enhancers, 2800 as typical enhancers, and 1615 as new or putative enhancers that were not previously known. FIREs for 456 disease-associated single nucleotide polymorphisms (SNPs) and quantitative trait loci (QTLs) were also identified. Distinct disease-associated FIREs were found in specific tissues or cell types, which further strengthens the association; for example, Alzheimer’s SNPs were found in brain-specific FIREs, and SNPs for acute lymphoblastic leukemia were found in GM12878-specific super-FIREs. The tissue and cell specificity of these interaction regions may help reveal how disease variants manifest in tissue-specific phenotypes.

CHi-C methods can substantially aid in connecting disease-associated SNPs with target genes, and have already advanced our understanding of the genetic basis for many diseases. CHi-C was first utilized to understand the interactions of three cancer risk-associated regions that lie in gene deserts. In this approach, 519 bait probes were used to capture regions interacting with these gene desert loci and identified interacting regions that included protein-coding genes, lncRNAs, and cancer-associated SNPs [82]. Similarly, CHi-C was also employed in defining interactomes for 14 colorectal-cancer-risk-associated loci [83]. These distal interacting regions of disease risk likely harbor regulatory elements that are altered and confer disease; therefore, this has prognostic potential by identifying causal variants.

A detailed catalogue of 22,000 promoter interactions was generated using promoter CHi-C for two blood cell types: the lymphoblast line GM12878 and CD34^+^ hematopoietic progenitor cells. 3D interaction data indicated that SNPs associated with autoimmune and other hematological disorders were significantly enriched at interacting distal regulatory sites of targeted promoters [84], suggesting that these genes are likely dysregulated in the disease state. Similarly, CHi-C was also applied to study autoimmune-disease associated SNPs in GM12878 B-cell and Jurkat T-cell lines. These findings demonstrated that different autoimmune-associated variants interact with common gene promoters, which are presumably dysregulated. They also contradict the long-held assumption that disease-causing genes and their associated variants should be in close linkage disequilibrium (LD) to impart an effect [85]. Another recent study leveraged existing Hi-C data to determine that variants at regulatory elements outside of LD blocks interacted with genes or their enhancers harboring linked SNPs to impact gene expression and disease risk [86]. These variants were termed “outside variants” based on their location outside of LD blocks.

Another study generated extensive catalogues of distal genomic regions that interact with promoters, or promoter-interacting regions (PIRs), in 17 primary hematopoietic cell types [7]. The authors linked 2500 novel SNPs to putative disease-associated genes related to blood and autoimmune disorders. It was observed that PIRs were highly cell type specific, and, as noted above, this implicates which variants are likely drivers of cell-specific phenotypes due to their location in either cell-specific enhancers or regulatory loops. Novel putative enhancers, which lacked typical enhancer chromatin signatures such as histone methylation, were identified through these captured interactions and showed an additive effect on gene expression. This additive effect indicates that more than one enhancer interacts on a particular target. This one-target–multi-enhancer model suggests the evolution of fail-safe transcriptional circuits, wherein mutation in one or two enhancers may not lead to the breakdown of target gene activation [7]. In support of this model, Temp enhancers were recently described in hESCs surrounding the *POU5F1* locus, which encodes OCT4, a master regulator of ESCs [60]. CRISPR-Cas9-targeted deletion of certain enhancers led to only a temporary reduction of the OCT4-encoding transcript, which eventually returned to normal levels. This could have important implications in understanding how epistatic relationships between *cis*-regulatory elements are used to maintain cellular homeostasis.

A central goal of modern genomics research is to translate GWAS discoveries into therapeutic outcomes. A CHi-C study of a critical autoimmune risk locus on chromosome 6q23 reported that different autoimmune related disorders such as rheumatoid arthritis, psoriasis, and type 1 diabetes were regulated by a common intergenic enhancer, suggesting a “transcription factory”-like structure. Moreover, the research identified the involvement of a novel gene, *IL20RA*, and suggested that treatment using monoclonal antibodies targeting its ligand IL20 may provide better therapeutic outcome for the related autoimmune disorders [87]. Importantly, autoimmune diseases, and similarly neurological disorders, often share disease-associated variants; thus, future investigation of looping structures could reveal common mechanisms for multiple diseases within these broader categories.

Finally, Hi-C can be used for genome assembly [88], which has important implications for the study of disease. For example, genome assembly is proving important in determining copy number variants and translocation in cancer, and may also have applications to neurological disorders and others in which chromosomal deletions, inversions, or duplications are drivers of the disease. It is being used to phase genomes for haplotype structures [89], which will aid our understanding of inherited alleles and their variants, relevant for rare inherited diseases. Hi-C is also being used for rapid genome assembly of pathogens [90], as the proximity ligation, on which the method relies, enables assembly without prior knowledge of genome sequence or structure. This is sure to influence how we understand host–pathogen interactions and treatments.

## Conclusions and future perspectives

The applications and development of technologies to investigate 3D genome architecture are rapidly changing how we view genomics. Advances in our understanding of architectural arrangements for precise loci using Hi-C, CHi-C, and allied techniques are helping to associate non-coding (regulatory) disease variants (SNPs) with the most probable target genes, and could explain pathomechanisms mediated via distal regulatory variants. Moreover, the identification of genes interacting with disease-associated regulatory variants provides a basis for determining enriched signaling pathways involved in the pathogenesis of diseases, which may lead to therapeutic interventions that are more readily targetable than those aimed at the variant or TF that binds the site. The recent application of CRISPR-based tools and integrated “C”-based techniques are likely to further our understanding of the relationship between regulation and 3D architecture.

Multiple genetic disorders, as discussed above, have been associated with altered architectural modules. Combining Hi-C data with genome-editing tools may lead to therapeutic outcomes via cell-based therapy and the introduction or removal of architectural modules. Similarly, site-specific epigenome editing has also paved the way for the investigation of directed architectural changes. Catalogues of interaction maps from various cells and tissues now serve as references for comparing future 3D genome maps from diseased states. Computational tools to facilitate analysis of these new datasets are concurrently being developed. Collectively, this will further the clinical applications of 3D genomics.

Increasing evidence indicates the strong possibility of transcription factory or hub-like structures in cells, in which multiple enhancers, silencers or other elements may regulate one or more promoters together [7, 66, 91]. Screening the complex epistatic interactions within these regulatory loops may uncover novel mechanisms of disease resulting from disrupted architectural regulation. In summary, we are beginning to learn if or how single-nucleotide and structural variants impact genome folding. The rapid technological advances in this field have highlighted the importance of studying 3D genomics to improve prognostic, diagnostic, and potentially therapeutic outcomes.

## Abbreviations

3C: Chromosome conformation capture
3D: Three-dimensional
4C: Circular chromosome conformation capture on chip
4C-seq: Circular chromosome conformation capture on chip combined with sequencing
5C: Chromosome conformation capture carbon copy
bp: Base pairs
capture-C: Chromosome conformation capture coupled with oligonucleotide capture technology
CBS: CTCF-binding site
CERES: CRISPR-Cas9-based epigenetic regulatory element screening
ChIA-PET: Chromatin interaction analysis-end tag sequencing
CHi-C: Hi-C coupled with RNA bait capture probes
ChIP-seq: chromatin immunoprecipitation followed by sequencing
CRISPR: Clustered regularly interspaced short palindromic repeats
CRISPRi: CRISPR interference
DHS: DNase I hypersensitive site
DNase Hi-C: Genome-wide chromatin conformation capture with DNase I digestion
FIREs: Frequently interacting enhancer regions
FISH: Fluorescence in situ hybridization
GAM: Genome architectural mapping
GWAS: Genome-wide association study
hESC: Human embryonic stem cell
Hi-C: Genome-wide chromatin conformation capture
Hi-Cap: Hi-C capture
HiChIP: Hi-C chromatin immunoprecipitation
kb: Kilobase
LAD: Lamina-associated domain
LD: Linkage disequilibrium
lincRNA: Long intergenic non-coding RNA
lncRNA: Long non-coding RNA
LTP: Long-term potentiation
Mb: Megabase
mESC: Mouse embryonic stem cell
NG-capture-C: Next-generation capture-C
PIR: Promoter-interacting regions
PLAC-seq: Proximity ligation assisted chromatin immunoprecipitation
QTL: Quantitative trait loci
RNA-seq: RNA sequencing
sciHi-C: Single-cell combinatorial indexing Hi-C
sgRNA: Single guide RNA
SNP: Single nucleotide polymorphism
SNV: Single nucleotide variant
T2C: Targeted chromatin capture
TAD: Topologically associated domain
TCC: Tethered conformation capture
Temp: Temporarily phenotypic
TF: Transcription factor
TLA: Targeted locus amplification
